# Complex coronary lesions: increasing safety and outcomes with optimal lesion preparation as well as prompt complication management

**DOI:** 10.1093/ehjcr/ytaf396

**Published:** 2025-08-23

**Authors:** Farhang Aminfar, David Meier, Vladimir Rubimbura

**Affiliations:** Department of Cardiology, Lausanne University Hospital and University of Lausanne, Rue du Bugnon 46, Lausanne 1011, Switzerland; Department of Cardiology, Lausanne University Hospital and University of Lausanne, Rue du Bugnon 46, Lausanne 1011, Switzerland; Department of Cardiology, Lausanne University Hospital and University of Lausanne, Rue du Bugnon 46, Lausanne 1011, Switzerland

We read with interest the comments by Çiloğlu *et al.*^1^ regarding our case report on late retrieval of an unnoticed stent loss.^2^ We appreciate the engagement and the opportunity to elaborate on several of the key points raised.

## Rationale for complete revascularization in complex ST-elevation myocardial infarction

Percutaneous coronary intervention (PCI) of non-culprit lesions has been shown to reduce the risk of cardiac events in patients presenting with ST-elevation myocardial infarction.^3^ However, in the context of complex coronary lesions, we agree that the benefits of revascularization must be carefully weighed against the procedural risks. Our decision to pursue complete revascularization was based on the absence of anterior wall motion abnormalities, the severity of the left anterior descending artery/diagonal bifurcation lesion, and the extensive myocardial territory at risk. In general, we favour a staged approach for complex lesions, given the associated risks and potential complications. Nevertheless, for very tight stenoses, we believe that performing PCI during the same hospital stay can reduce the risk of unplanned revascularization or subsequent myocardial infarction related to the non-culprit lesion.^3^

## Role of intravascular imaging and lesion preparation

Intracoronary imaging has become essential in the management of complex and calcified coronary lesions, with a Class IA recommendation in current clinical guidelines.^4,5^ It provides invaluable insights into plaque morphology, calcium burden, and the adequacy of lesion modification, thereby guiding the use of adjunctive techniques like rotational atherectomy or intravascular lithotripsy.^5,6^ Post-PCI, imaging also ensures optimal stent expansion, which is crucial for favourable long-term outcomes.^5^ In our case, intravascular imaging was deferred during the initial intervention due to a high contrast load and a septal perforation, but was employed during the follow-up procedure. We acknowledge that earlier imaging might have prompted more aggressive plaque modification and potentially prevented stent loss, as highlighted by Dr Güner’s team.^1^

Regarding lesion preparation, we are convinced that optimal plaque modification is essential prior to stenting. The balloon’s apparent complete expansion falsely reassured us regarding adequate lesion preparation. We believe that more aggressive calcium modification, guided by intracoronary imaging, might have facilitated stent delivery. Overall, our case^2^ highlights the importance of optimal strategy selection and adequate lesion preparation, as emphasized in the recent EAPCI and EURO4C-PCR consensus document^6^ that offers practical guidance to optimize procedural outcomes.

## Prevention and management of stent loss

We agree that preventing stent loss hinges on meticulous lesion preparation. As previously noted,^2^ systematically inspecting the stent delivery system after failed deployment is essential for early detection. In our case, simultaneous management of a coronary perforation contributed to the oversight. The subsequent partial endothelialization of the lost stent complicated retrieval, resulting in dissection and necessitating further stenting. Intracoronary imaging and dedicated retrieval devices are vital for managing such complications.

## Conclusion

**Figure 1:**
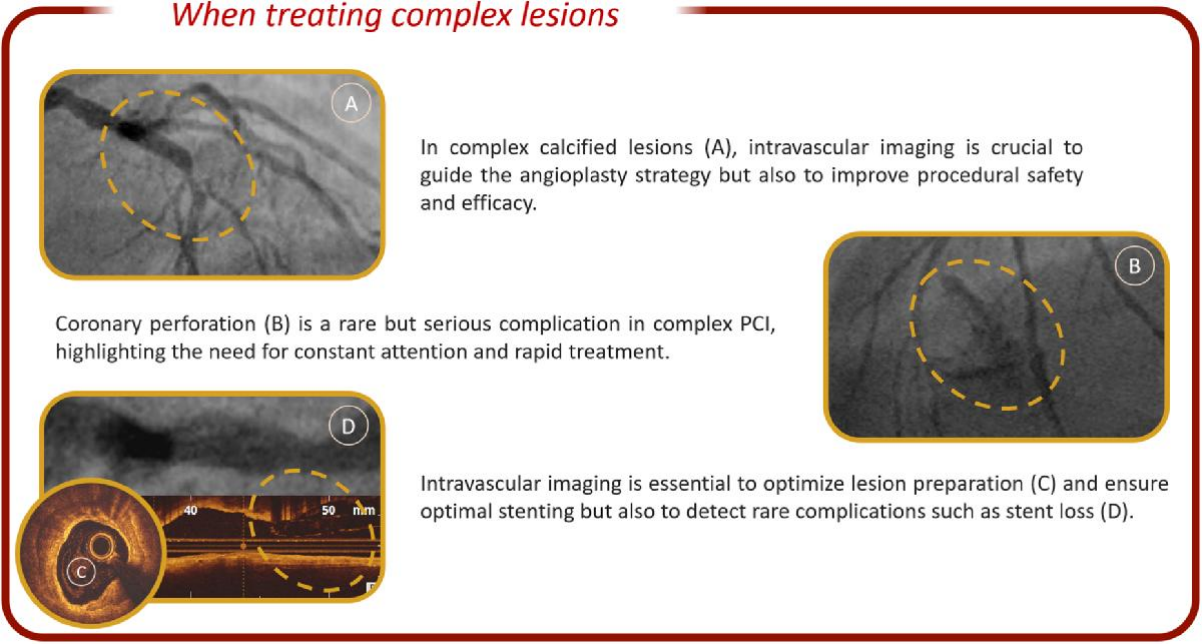
What should be remembered when treating complex and calcified coronary lesions in order to improve procedural safety and outcomes.

In summary, we emphasize the importance of individualized revascularization strategies in STEMI with complex lesions, the critical role of intravascular imaging and adequate lesion preparation, and the importance of vigilance in preventing and managing rare complications such as stent loss (*Figure 1*).

## Data Availability

The data underlying this article are available in the article and in its online supplementary material.
